# Transmembrane protein 14A protects glomerular filtration barrier integrity

**DOI:** 10.14814/phy2.15847

**Published:** 2023-12-06

**Authors:** Ramzi Khalil, Josephine D. D. Bonnemaijer, Reinhold Kreutz, Herman P. Spaink, Pancras C. W. Hogendoorn, Hans J. Baelde

**Affiliations:** ^1^ Department of Pathology Leiden University Medical Center Leiden The Netherlands; ^2^ Institute of Clinical Pharmacology and Toxicology Charité ‐ University Medicine Berlin Germany; ^3^ Institute of Biology Leiden Leiden University Leiden The Netherlands

**Keywords:** cell, glomerulus, kidney, podocyte, proteinuria, rat, TMEM14A, zebrafish

## Abstract

Transmembrane protein 14A (TMEM14A) is a relatively unknown protein that is now identified to be required for maintaining the integrity of the glomerular filtration barrier. It is an integral transmembrane protein of 99 amino acids with three transmembrane domains. TMEM14A has been implied to suppress Bax‐mediated apoptosis in other studies. Other than that, little is currently known of its function. Here, we show that its expression is diminished before onset of proteinuria in a spontaneously proteinuric rat model. Knocking down *tmem14a* mRNA translation results in proteinuria in zebrafish embryos without affecting tubular reabsorption. Also, it is primarily expressed by podocytes. Lastly, an increase in glomerular *TMEM14A* expression is exhibited in various proteinuric renal diseases. Overall, these results suggest that TMEM14A is a novel factor in the protective mechanisms of the nephron to maintain glomerular filtration barrier integrity.

## 1 INTRODUCTION

Proteinuria is an important risk factor for progression of renal disease and cardiovascular mortality (Group, 2013). Proteinuria occurs when glomerular filtration barrier (GFB) integrity is compromised. The GFB consists of fenestrated endothelial cells lined with glycocalyx, the glomerular basement membrane, and podocyte foot processes. Understanding the pathophysiology and mechanisms leading to proteinuria is essential in the quest to understanding nephron function and finding new potential therapeutic targets for proteinuric renal diseases.

To study the pathophysiology and underlying mechanisms of the development of proteinuria, we have further analyzed spontaneously proteinuric Dahl salt sensitive rats (Dahl) (Garrett et al., 2003, 2006; Khalil, Koop, et al., 2019; Mehr et al., 2003; Siegel et al., 2004). Analysis of differentially regulated glomerular mRNA yielded various genes potentially involved in the development of proteinuria (Khalil, Koop, et al., 2019; Koop et al., 2008). One of the noteworthy downregulated genes in the Dahl rat, was transmembrane protein 14A (Tmem14a).

TMEM14A is 99 amino acid integral membrane protein with three transmembrane domains. Its structure has been identified by nuclear magnetic resonance spectroscopy (Klammt et al., 2012). Relatively little is known of its function. It has been described to be involved in preventing apoptosis by preventing loss of mitochondrial membrane potential through Bax suppression in an in vitro study (Woo et al., 2011). However, its function in maintenance of GFB integrity has not yet been described. Interestingly, apoptosis of podocytes has been described as a pathophysiological process in proteinuric renal disease, especially diabetic nephropathy, in various experimental models (Cardoso et al., 2018; Lee et al., 2009; Tao et al., 2023; Zhou et al., 2009, 2012). Moreover, podocyte detachment and loss has been suggested to been dependent on apoptotic caspases in an experimental animal model (Yamamoto et al., 2022).

As *Tmem14a* was found to be downregulated in rats with a proteinuric phenotype in the Dahl array, we hypothesize that it is required for maintaining GFB integrity and that is also differentially expressed in human proteinuric disease.

## 2 MATERIALS AND METHODS

### 2.1 Microarray

Microarray data containing information on differentially regulated genes in glomeruli obtained from spontaneously proteinuric Dahl salt sensitive (Dahl) male rats strain and non‐proteinuric spontaneous hypertensive (SHR) male rats at 4 and 6 weeks of age was used to identify genes potentially involved in the development of proteinuria. The dataset with GEO Series accession number GSE 13810 was accessed through the Gene Expression Omnibus of the NCBI. As described previously, this analysis was performed with Affymetrix GeneChip Rat Genome 230 2.0 arrays (Khalil, Koop, et al., 2019).

### 2.2 Animal studies: Rats

Animal studies in rats were performed with the same animal material as previously described and in accordance with institutional guidelines (Khalil, Koop, et al., 2019; Koop et al., 2008). Rat experiments were approved by the respective Institutional Animal Care and Use Committee. Dahl and SHR male rats were obtained from Freie Universität Berlin (Mehr et al., 2003). They were fed a low salt diet containing 0.2 percent NaCl by weight to prevent early development of hypertension. To investigate the time relation between Tmem14a expression and the development of proteinuria, groups of rats were studied at 2, 4, 6, 8, and 10 weeks of age. mRNA expression was investigated in a total of 67 animals. For the respective ages stated above the respective number of investigated animals per age group were 7, 7, 7, 8, and 8 for the Dahl rats and a number of 5, 4, 6, 7, and 8 for the SHR group. For protein expression, two rats were investigated per age and group.

### 2.3 Animal studies: Zebrafish

Wild‐type (WT) AB/TL strain zebrafish (*Danio rerio H)* were injected with 1 nL of a morpholino (Gene Tools, Philomath, OR) blocking the mRNA translation of the zebrafish *tmem14a* homologue, zgc:163080, or a scrambled control morpholino during the 1‐to‐4 cell stage of embryonic development (Kimmel et al., 1995). Experiments were performed concurrently with previously described studies (Khalil, Koop, et al., 2019; Khalil, Lalai, et al., 2019).

All zebrafish experiments were performed prior to the free‐feeding stage of embryos and therefore are not considered animal experiments in accordance with EU Animal Protection Directive 2010/63/EU.

### 2.4 Glomerular permeability and tubular reabsorption assay

A tubular reabsorption assay was performed as described previously (Elmonem et al., 2017; Khalil, Koop, et al., 2019; Khalil, Lalai, et al., 2019). This method was adapted from Hentschel et al. and has been previously validated to reliably assess glomerular permeability and tubular reabsorption function (Hanke et al., 2013; Hentschel et al., 2007). In short, a 1 nL mixture of 3 kDa TRITC labeled dextran (100 mg/mL; Invitrogen) and 70 kDa FITC labeled dextran (25 mg/mL; Invitrogen) was injected intravenously at 5 days post fertilization (dpf), fixed 1 h after injection in 10% formalin for 24 h, stored in ethanol 70%, embedded in paraffin, sectioned, and examined by fluorescence microscopy. The number of proximal tubule reabsorption droplets were quantified manually in a blinded fashion. As a positive control, a section of control zebrafish embryos was injected with puromycin aminonucleoside (PAN, Sigma‐Aldrich, St. Louis, MO) at 4 dpf (Hentschel et al., 2007). Under physiological conditions, the 3 kDa tracer freely passes the GFB and the 70 kDa does not. After passing the GFB, these tracers are absorbed by proximal tubule cells in endosomes, appearing as small droplets. Hence, observing 70 kDa reabsorption droplets in proximal tubule cells indicates loss of GFB integrity. Reabsorption droplets of the 3‐kDa tracer were used to assess the correct location of the proximal tubule cells and whether tubular reabsorption mechanisms are sufficiently intact.

Although injecting dextrans of sizes larger than 70 kDa could theoretically provide additional information on the severity of loss of GFB integrity, previous pilot experiments (data not shown) have shown 70 kDa to produce the most consistent and reliable results when fixing the samples 1 h after injection. We were not able to reliably differentiate between the 70 kDa and larger tracers when maintaining this timeframe. Therefore, in this study, only the validated 3‐ and 70 kDa tracers were used.

### 2.5 Cell culture

Immortalized podocytes (Moin Saleem, Bristol, UK, human), embryonic kidney (HEK) 293 cells (ATCC), and human umbilical vein endothelial cells (Huvec) (Lonza, Allendale, NJ, USA) ATCC were used to measure mRNA expression for *TMEM14A*. Podocytes were also used for localization of TMEM14A protein expression by immunohistochemistry as described below. Immortalized podocytes were cultured in RPMI 1640 Medium with added Penicillin, Streptomycin, Insulin, Transferrin, Selenite (Sigma Chemicals, Dorset, UK) and 10% fetal bovine serum at 33°C (in 5% CO_2_) for proliferation and at 37°C (in 5% CO_2_) for differentiation.

### 2.6 RNA isolation, reverse transcription, and qPCR

RNA isolation, reverse transcription, and qPCR were performed as described previously (Khalil, Koop, et al., 2019). To quantify gene expression of *TMEM14A* in different cell lines, RNA from podocytes, HEK, and Huvec cells, and RNA from purified glomeruli and whole kidney tissue was isolated using TRIzol (Invitrogen, Waltham, MA). AMV reverse transcriptase (Roche Diagnostics) was used for reverse transcription into cDNA. Primer sequences are listed in Table 1. GAPDH was used as a housekeeping gene for cell culture experiments. For purified rat glomeruli experiments, *Hprt1* was used as internal control. mRNA expression values are given as relative to *Hprt1* expression. Quantitative real‐time PCR (qPCR) was performed on an iCycler real‐time PCR machine with SYBR green supermix. (Bio‐Rad Laboratories, Hercules, CA), and Bio‐Rad CFX Maestro software was used for normalized gene expression calculations. (Bio‐Rad Laboratories).

**TABLE 1:** Primer sequences.

Name	Symbol	mRNA sequence	Forward primer Reverse primer
Transmembrane protein 14A (human)	TMEM14A	NM_014051.3	TTTGGTTATGCAGCCCTCGT ATAGCCGGCCAAACATCCAA
Glyceraldehyde‐3‐phosphate dehydrogenase (human)	GAPDH	NM_002046	TGGTCACCAGGGCTGCTT AGCTTCCCGTTCTCAGCCTT
Hypoxanthine‐guanine phosphoribosyltransferase 1 (human)	HPRT1	NM_000194.2	TGACACTGGCAAAACAATGCA GGTCCTTTTCACCAGCAAGCT
Transmembrane protein 14A (rat)	*Tmem14a*	NM_014051.4	GGCCACCATAATGGGTGTGA CAGCAGCAGGACAAGTCTCA
Hypoxanthine‐guanine phosphoribosyltransferase 1 (rat)	*Hprt1*	NM_012583.2	GGCTATAAGTTCTTTGCTGACCTG AACTTTTATGTCCCCCGTTGA

### 2.7 Human material

Anonymized tissue from renal biopsies from patients with various proteinuric renal diseases was collected from a previously described archive, including 20 patients with IgA nephropathy, 20 patients with lupus nephritis class III or IV, 10 patients with minimal change disease (MCD), and 13 patients with diabetes nephropathy (DN) at the department of pathology, Leiden University Medical Center (Khalil, Koop, et al., 2019). Tissue of 10 kidneys that were unsuitable for transplantation and tumor‐free renal resection material of 10 patients with a tumor elsewhere in the material were collected and used as a control group. All the materials were obtained, anonymized, and handled according to institutional guidelines, Good Research Practice, and the Code of conduct for responsible use.

### 2.8 Immunohistochemistry

A commercially available polyclonal goat anti‐TMEM14A antibody (Santa Cruz Biotech, sc‐248,899, Dallas, TX) was used for immunohistochemical staining of TMEM14A on human and rat material. In zebrafish material, the quality of staining was insufficient for reliable assessment.

Immortalized podocytes that were confluent for 2 weeks were transferred to small glasses in a 24‐well plate. After 24 h incubation immunohistochemistry was performed. Podocytes were washed in PBS and fixated using –20°C methanol. The glasses were then washed in PBS and blocked with 5% Normal Rabbit Serum (NRS) for 1 h. 5% NRS was aspirated, and the glasses were incubated with primary antibody diluted in 1% BSA in PBS (polyclonal goat anti‐TMEM14A) at 4°C overnight.

For rat and human kidney tissue, after sectioning at 4 μm, the slides were deparaffinized, dehydrated, and boiled in Tris/EDTA buffer for antigen retrieval for 10 min. The slides were washed in PBS and incubated with the primary antibody diluted in 1% BSA in PBS (polyclonal goat anti‐TMEM14A 1:200 for rat tissue, 1:150 for human tissue) at 4°C overnight.

For all mentioned materials, after incubation of the primary antibody the samples were washed in PBS and incubated for 30 min with the secondary antibody (Polyclonal Rabbit Anti‐Goat Immunoglobulins/horse radish peroxidase, Cat no: P0160, AgilentDako, CA, United States).The material was then washed in PBS again and immunoreactivity was detected with diaminobenzidine. After counterstaining with hematoxylin, the material was dehydrated and mounted.

For quantification of stained slides in both rat and human material, we used a semiquantitative approach. TMEM14A staining was evaluated on a semiquantitative scale with a score of 0–4 in a blinded manner, subdivided in respectively no podocyte staining (0), 0%–10% of the podocytes (1), 10%–30% of the podocytes (2), 30%–60% of the podocytes (3), and more than 60% of the podocytes (4).

### 2.9 Statistical analyses

Statistical analyses were performed using GraphPad Prism 9.4.1 (GraphPad Software, San Diego, California). Student's unpaired *t*‐testing was used for comparisons between two or three groups. When more than three groups were compared, one‐way ANOVA with Tukey's post hoc analysis was used. A *p*‐value below 0.05 was considered significant.

## 3 RESULTS

### 3.1 Glomerular gene expression pattern of proteinuric rats

*Tmem14a* was identified as potentially involved in the development of proteinuria through analysis of a previously published microarray dataset comparing Dahl and SHR rats. The top five downregulated genes are shown in Table 2. These genes are found in both SHR and Dahl rats. *Tmem14a* was selected for further analysis based on this differential expression and the availability of a zebrafish homologue to perform experimental in vivo analysis of development of proteinuria, as performed later in this study.

**TABLE 2:** Glomerular gene expression in Dahl rats compared to SHR.

Gene name	Symbol	Region of rat chromosome	Fold change
Aldo‐keto reductase family 1, member B8	Akr1b8	4q22	−4.5
Similar to interferon regulatory factor 10	RGD1562711	3q41	−3.9
Acyl‐Coenzyme A oxidase 2, branched chain	Acox2	15p14	−3.7
Similar to RIKEN cDNA 4921520P21; DMRTC1	LOC363483	Xq31	−3.4
Transmembrane protein 14A	Tmem14a	9q13	−3.0

### 3.2 Glomerular Tmem14a mRNA and protein expression is diminished in spontaneously proteinuric rats before onset of proteinuria

**FIGURE 1:**
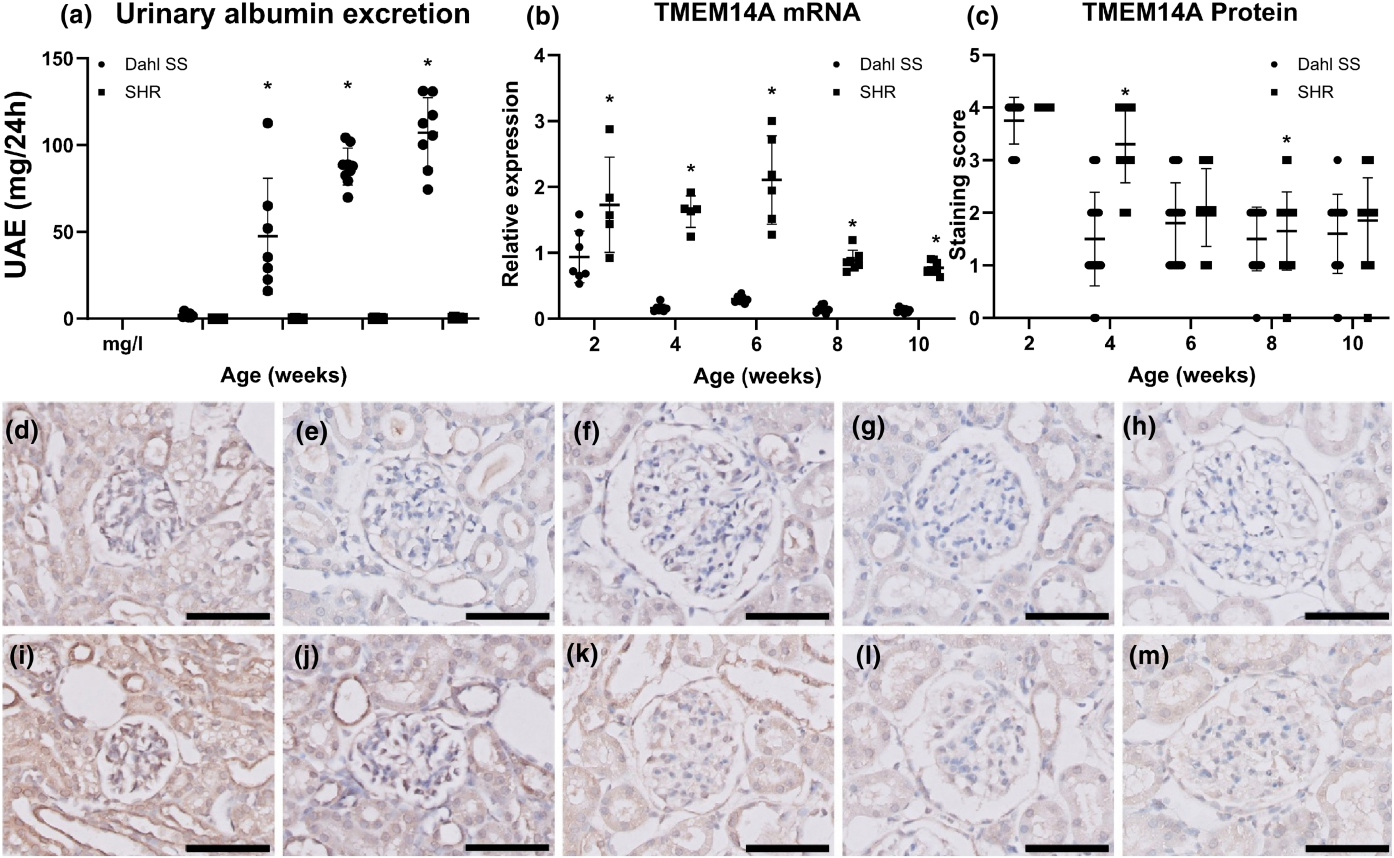
Glomerular TMEM14A expression was diminished before onset of proteinuria. (a) Urinary albumin excretion is significantly higher in Dahl rats compared to SHR from 6 weeks of age. Y‐axis shows urinary albumin excretion in mg/24 h, and the X‐axis shows animal age in weeks. (b) Glomerular TMEM14A mRNA expression in Dahl rats is significantly lower than in SHR controls at all time points. The Y‐axis shows mRNA expression as relative expression compared to *Hprt1*, and the X‐axis shows animal age in weeks. No correlation was observed between TMEM14A expression and urinary albumin excretion in both SHR (*r* = − 0.80, CI −0.98 to 0.27) and Dahl (*r* = −0.60, CI −0.97 to 0.60) using Pearson's correlation coefficient. (c) Glomerular staining for Tmem14a protein is lower in Dahl rats than in SHR after 2 weeks. This difference is significant at Weeks 4 and 8 of age. The columns represent mean semiquantitative score. Dahl rats develop proteinuria at 6 weeks of age. Thus, both mRNA and protein expression levels drop before onset of proteinuria. ANOVA with Tukey's post hoc analysis was used.(d–m) Representative images of glomeruli of spontaneously proteinuric Dahl (d–h) and SHR (i–m) rats at respectively 2 (d and i), 4 (e and j), 6 (f and k), 8 (g and l), and 10 (h and m) weeks of age. (a–c) Horizontal lines indicate mean with SD. Scale bar = 50 μm *p < 0.001.

Glomerular mRNA and protein expression of Tmem14a were investigated in Dahl rats and compared to spontaneously hypertensive rats at 2, 4, 6, 8, and 10 weeks of age. Of note, Dahl rats develop significant proteinuria from 6 weeks of age. (Figure 1a) When comparing the two groups at each time point, glomerular *Tmem14a* mRNA expression is consistently significantly lower in the Dahl rats (*p* < 0.0001 at every time point, Figure 1a). Looking at the difference in expression between the different time points in only the Dahl rats, expression starts high and decreases quickly; at 2 weeks of age expression is significantly higher than at all other time points (*p* < 0.0001), but no significant difference was seen between the other time points. Doing the same in the spontaneously hypertensive control, glomerular *Tmem14a* mRNA expression was significantly higher at a younger age at Weeks 2, 4, and 6 compared to Week 10 (*p* < 0.01, *p* < 0.05, and *p* < 0.0001, respectively). At Weeks 2, 4, and 6, expression was significantly higher than at 8 weeks of age (*p* < 0.05, *p* < 0.05, and *p* < 0.001, respectively).

After 2 weeks of age Tmem14a protein expression was lower in Dahl rats than in SHR at all time points. This was significantly so at 4 and 8 weeks of age (*p* < 0.001). At later time points, no significant differences in glomerular Tmem14a protein expression were seen. So, in Dahl rats, glomerular Tmem14a mRNA and protein expression is diminished before onset of proteinuria.

### 3.3 Knocking down tmem14a mRNA translation results in proteinuria in zebrafish embryos

**FIGURE 2:**
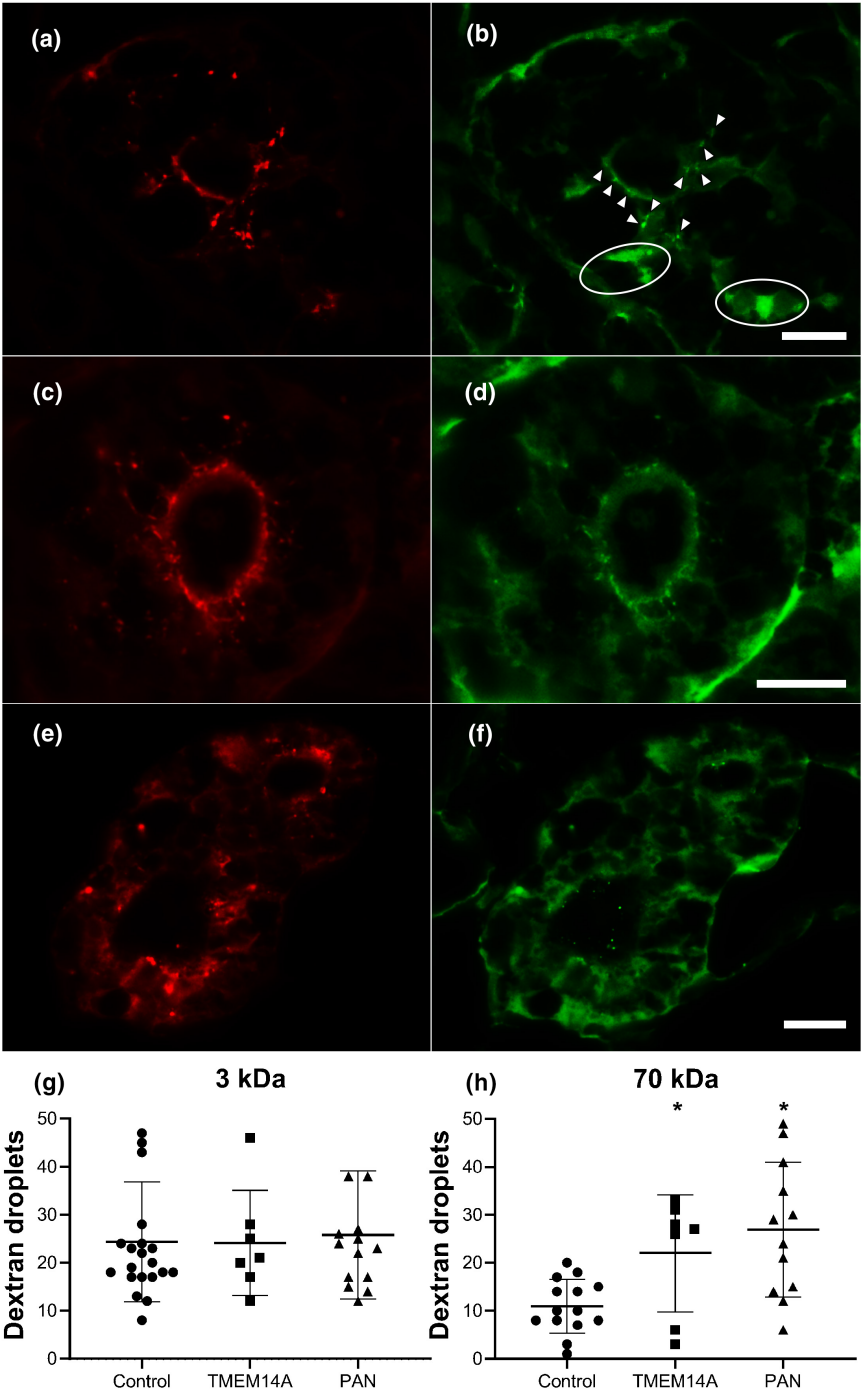
Knocking down TMEM14A mRNA translation causes proteinuria. Knocking down mRNA translation of the zebrafish homologue of TMEM14A through morpholino injection results in proteinuria. (a–f) Representative immunofluorescence images of transversal sections of zebrafish proximal tubule cells after injection of a mixture of red labeled 3 kDa dextran tracer (a, c, e) and green labeled 70 kDa dextran tracer (b, d, f) in controls (a,b), TMEM14A knockdowns (C and d), and PAN injected positive controls (e,f). Dextran tracers that passed the GFB are reabsorbed by proximal tubule epithelial cells in endosomes. Thus, reabsorbed dextran tracer appears as fluorescent droplets. The number of proximal tubule reabsorption droplets was counted in a blinded manner in sections as those shown here. The arrowheads in B point out examples of counted droplets. The circled areas show high fluorescence due to dextran present in the peritubular capillaries. These areas are not counted as reabsorption droplets. The sharpness of the images in panels A through F has been enhanced by overlaying them with a digital high pass filter. (g and h) Uptake of the red 3 kDa marker (g) was used to assess tubular reabsorption function, which was intact in both TMEM14A knockdown animals and controls. In the knockdown model, significantly more 70 kDa droplets (h) have passed the GFB and were subsequently reabsorbed. Puromycin aminonucleoside (PAN) injected zebrafish were used as positive controls. Students *t*‐test was used.(g and h) Horizontal lines indicate mean with SD. Scale bar = 20 μm.* = *p* < 0.05.

The functional role of TMEM14A in the development of proteinuria was further investigated using a zebrafish embryo model. First, the zebrafish homologue of TMEM14A, zgc:163080, was knocked down by blocking its mRNA translation through morpholino injection. Then, a mixture of 3 and 70 kDa dextran tracers was injected. Puromycin aminonucleoside (PAN) injected zebrafish were used as a positive control for inducing proteinuria. PAN is a validated method to induce proteinuria in this zebrafish embryo model (Hanke et al., 2013; Hentschel et al., 2007). If the injected dextran tracers pass the GFB, they are subsequently reabsorbed by proximal tubular epithelial cells. These cells reabsorb the dextran tracers in endosomes. Through immunofluorescence microscopy, these endosomes appear as small fluorescent droplets in the proximal tubule cells. The number of these proximal tubule reabsorption droplets were counted in a blinded fashion. (Figures 2a through f) No difference was found in the mean number of 3 kDa reabsorption droplets, indicating that tubular reabsorption mechanisms remained intact in all three groups (Figure 2g). A significantly higher number of 70 kDa reabsorption droplets was measured in both zebrafish embryos with a *tmem14a* knockdown and in the positive control group, indicating loss of GFB integrity (Figure 2h, *p* < 0.05). The effectivity of *tmem14a* knockdown in zebrafish was primarily assessed by quantifying its effect on glomerular permeability.

### 3.4 TMEM14A is primarily expressed by podocytes in the kidney

**FIGURE 3:**
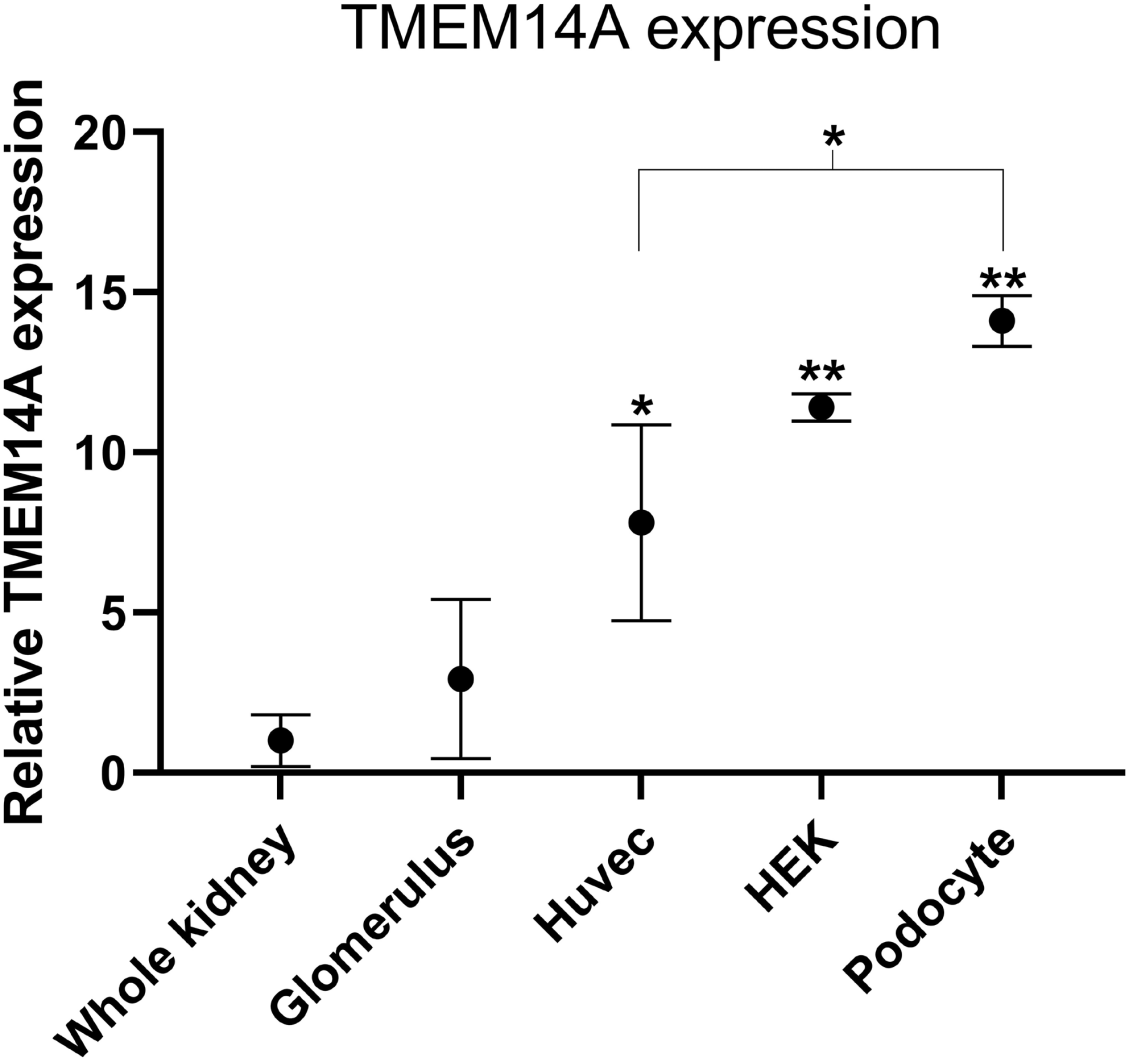
TMEM14A was primarily expressed by podocytes. In vitro experiments of relative TMEM14A mRNA expression show expression relative to GAPDH expression when comparing mRNA extracts from whole kidney, purified glomeruli, human umbilical vein endothelial cells (Huvec), human embryonic kidney (HEK), and finally podocytes. Expression in podocytes, HEK and Huvec was significantly higher than in whole kidney (*p* < 0.001 for all groups) and then purified glomeruli (*p* < 0.001 for podocytes and HEK, *p < 0.05* for Huvec). Podocyte expression was highest of all cells and significantly so compared to Huvec (*p* < 0.05). Mean and SD shown. ANOVA with Tukey's post hoc analysis was used.

*TMEM14A* mRNA expression was investigated—in both whole kidney tissue and purified glomeruli and compared to immortalized podocytes, HEK, and HUVEK cells. It revealed that *TMEM14A* is expressed by podocytes and endothelial cells. Moreover, its expression is higher in purified glomeruli than in whole kidney. mRNA expression was highest in differentiated podocytes (Figure 3a). Immunohistochemistry results from the previous experiments show that TMEM14A is also expressed in distal tubular cells (images 1c–k).

### 3.5 Glomerular TMEM14A protein expression is increased in proteinuric disease

**FIGURE 4:**
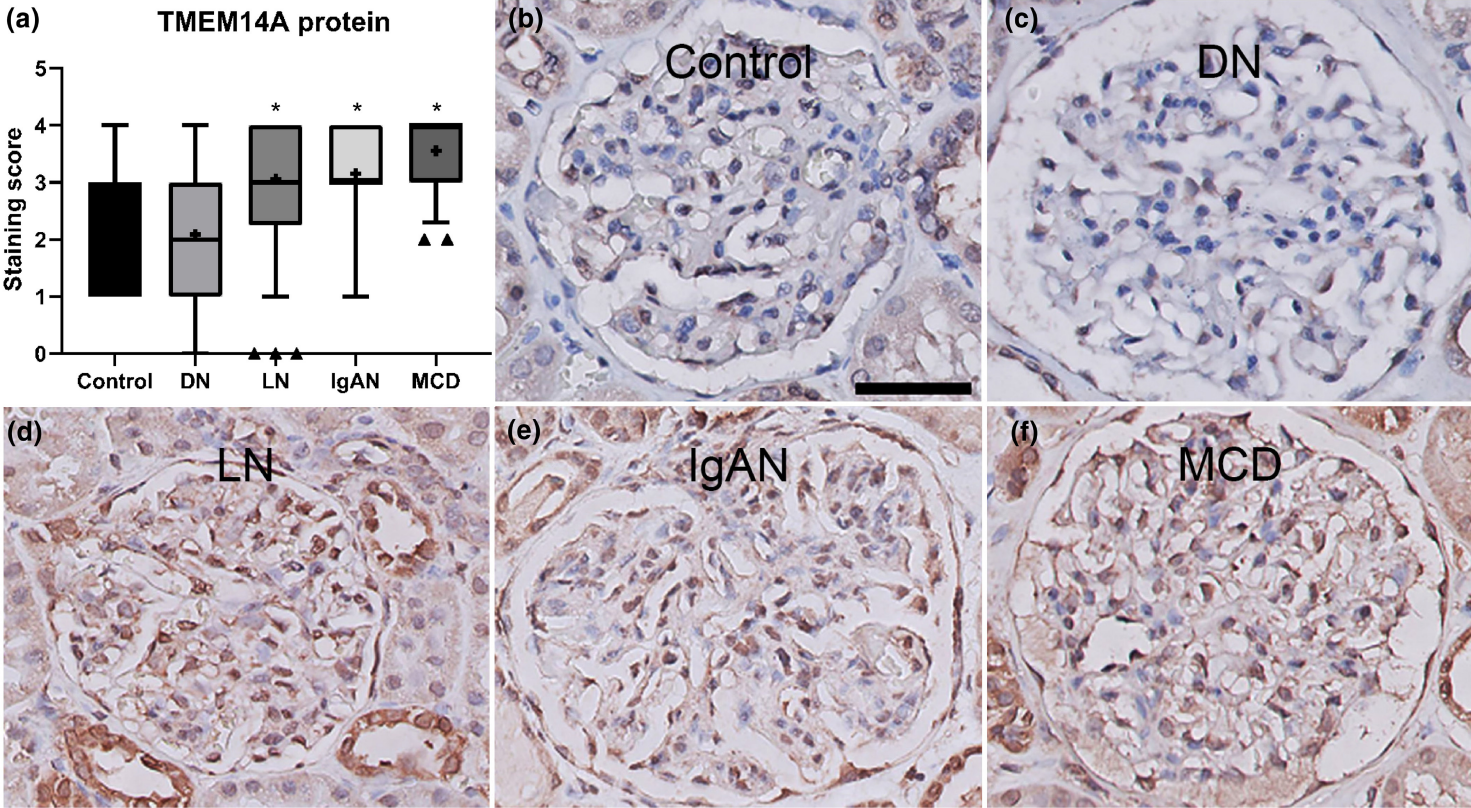
Glomerular TMEM14A expression was increased in human proteinuric renal diseases. (a) Glomerular TMEM14A protein expression was examined in human kidney biopsies from patients with diabetic nephropathy (DN), lupus nephritis (LN), IgA nephropathy (IgAN), minimal change disease (MCD), and healthy controls. Compared to controls, TMEM14A protein expression is significantly more extensive in IgAN, LN, and MCD, but not in DN. (b–f) Representative images of glomeruli stained for TMEM14A in healthy controls (b), diabetic nephropathy (c), lupus nephritis (d), IgA nephropathy (e), and minimal change disease (f). Slides were stained with goat anti‐TMEM14A antibody and immunoreactivity was assessed by diaminobenzidine. This results in a brown color which then indicates TMEM14A localization. Counterstaining with hematoxylin results in blue‐purple coloring of cell nuclei. Boxes in a show the range of values between the lower and upper quartile, the whiskers show 5th–95th percentile, triangles show values lying outside the 5th–95th percentile, the line in the box shows the median, and “+” indicates the mean. The scale bar in B applies to B through f and indicates 50 μm. * = p < 0.001. ANOVA with Tukey's post hoc analysis was used.

To establish whether TMEM14A also plays a role in the development of proteinuria in human disease, glomerular TMEM14A protein expression was examined in human kidney biopsies from patients with IgA nephropathy (IgAN), lupus nephritis (LN), minimal change disease (MCD), or diabetic nephropathy (DN). Here, we found that TMEM14A protein expression is significantly increased in IgAN, LN, and MCD (*p* < 0.0001) but not in DN (Figure 4). No correlation was found between the level of proteinuria and the score in TMEM14A positivity (*r* = 0.09).

## 4 DISCUSSION

TMEM14A is a relatively unknown protein that is identified to be essential in maintaining GFB integrity. In spontaneously proteinuric rats, we show that glomerular Tmem14a mRNA and protein expression is relatively diminished, especially before onset of proteinuria. Also, knocking down *tmem14a* mRNA translation in zebrafish results in proteinuria. In vitro experiments reveal that *TMEM14A* is primarily expressed by podocytes. Lastly, we show that glomerular TMEM14A protein expression is increased in various proteinuric renal diseases, but not in diabetic nephropathy.

Overall, these results imply that TMEM14A is an important factor in the development of proteinuria. Based on the results in this study, we postulate that its role in GFB integrity appears to be a protective one. First, a lack of sufficient *Tmem14a* expression in spontaneously proteinuric rats before onset of proteinuria supports this notion. Furthermore, direct inhibition of *tmem14a* mRNA translation resulted in proteinuria in the zebrafish model.

The specific function of the TMEM14A protein is not yet fully understood. As stated before, it has been associated with preventing Bax‐mediated apoptosis (Woo et al., 2011). Apoptosis was previously deemed to not be a significant cause of podocyte loss in most proteinuric diseases (Nagata, 2016). However, recent experiments by Yamamoto et al. show that loss of podocytes seems to be initiated by the start of the apoptotic process by caspase 3 (Yamamoto et al., 2022). Apoptosis pathways have previously been implicated as one of the pathophysiological mechanisms in the development of diabetic nephropathy and can be attenuated by ACE or ARBII inhibition (Cardoso et al., 2018; Lee et al., 2009). As no increase in glomerular TMEM14A protein expressing surface area was seen in diabetic nephropathy glomeruli, insufficient prevention of apoptosis due to a pre‐existent relative lack or impairment of TMEM14A might be a factor in the development of proteinuria in these patients. This would be in line with recent results from other studies suggesting that induction of apoptosis causes podocyte detachment (Yamamoto et al., 2022). Interestingly, previous studies show that Dahl rats indeed have a lower number of podocytes compared to SHR (Koop et al., 2008). The difference in TMEM14A expression between DN and other investigated proteinuric renal disease warrants further exploration, specifically into TMEM14A and apoptosis in diabetic nephropathy models.

The main limitation of this study is that is largely descriptive in nature, where the exact function of TMEM14A is not yet known. Additionally, external factors influencing its potential expression, degradation, and activation are not yet known and as such not yet investigated.

Also, although the use of various experimental models and species in this study is one of its strengths, it also provides a potential limitation due to interspecies variability, which might lead to an incomplete or incorrect analysis and extrapolation of results. As TMEM14A has strong homology across species, it could very well be an evolutionary well‐conserved gene that is part of a biological mechanism with broader involvement than the nephron alone.

Future studies are needed to further elucidate the exact role and surrounding mechanisms of TMEM14A in nephron function. In particular, interactions with other proteins involved in maintaining GFB integrity are yet to be unraveled. Moreover, the effect of knocking down TMEM14A through morpholino injection could be due to off target effect of morpholino injection. (Kok et al., 2015) The used anti‐TMEM14A antibody did not provide reliable assessment of TMEM14A expression in zebrafish and as such, it is possible that the observed proteinuria is an off target effect. It has been suggested in literature to validate observed morpholino‐induced phenotypes in embryos bearing mutations in the respective gene. The generation of a TMEM14A mutant would not only establish whether its role is indeed essential in the development of proteinuria, but also allow for additional mechanistic studies to its function.

## AUTHOR CONTRIBUTIONS

RKh conceived and designed research, performed experiments, analyzed data, interpreted results of experiments, prepared figures, drafted, edited, and revised the manuscript. JB performed experiments, analyzed data, interpreted results of experiments, prepared figures, and approved final version of manuscript. RKr and HS conceived and designed research, interpreted results of experiments, and approved final version of the manuscript. PH conceived and designed research, interpreted results of experiments, edited and revised manuscript, and approved final version of the manuscript. HB conceived and designed research, performed experiments, analyzed data, interpreted results of experiments, edited and revised manuscript, and approved final version of the manuscript.

## FUNDING INFORMATION

This work was supported in part by the Dutch National Kidney Foundation (IP 11.57).

## CONFLICT OF INTEREST STATEMENT

The authors declare that the research was conducted in the absence of any commercial or financial relationships that could be construed as a potential conflict of interest.

## 5 ETHICS STATEMENT

Rat experiments were approved by the respective Institutional Animal Care and Use Committee. All zebrafish experiments were performed prior to the free‐feeding stage of embryos and therefore are not considered animal experiments in accordance with EU Animal Protection Directive 2010/63/EU. All human renal biopsy tissue materials were obtained, anonymized, and handled according to institutional guidelines, Good Research Practice, and the Code of conduct for responsible use.

## Data Availability

Micro array data analyzed in this study are deposited and accessible at the Gene Expression Omnibus of the NCBI, which is accessible using the GEO Series accession number GSE 13810.
